# Do Dominant Native Ants Outcompete the Invasive Argentine Ant in Mediterranean Citrus Ecosystems? A Laboratory Test

**DOI:** 10.3390/insects15050333

**Published:** 2024-05-06

**Authors:** Vera Zina, Sofia Conde, Manuela Branco, José Carlos Franco

**Affiliations:** 1 Forest Research Centre, School of Agriculture, University of Lisbon, Tapada da Ajuda, 1349-017 Lisbon, Portugal; sofiaconde@isa.ulisboa.pt (S.C.); mrbranco@isa.ulisboa.pt (M.B.); jsantossilva@isa.ulisboa.pt (J.C.F.); 2 Laboratory for Sustainable Land Use and Ecosystem Services, School of Agriculture, University of Lisbon, Tapada da Ajuda, 1349-017 Lisbon, Portugal; 3 Agroscope, Research Division Agroecology and Environment, Reckenholzstrasse 191, 8046 Zurich, Switzerland

**Keywords:** dominance, Formicidae, interspecific competition, *Lasius grandis*, Mediterranean, *Tapinoma nigerrimum*

## Abstract

**Simple Summary:**

Invasive Argentine ants threaten Mediterranean citrus ecosystems. We investigated how native ants, like *Tapinoma nigerrimum* and *Lasius grandis*, compete with Argentine ants. Our experiments showed that native ants, with their larger size and unique defences, outperformed Argentine ants in individual confrontations. At the colony level, *Tapinoma nigerrimum* effectively defended food resources against Argentine ants. These findings suggest that native ants play a crucial role in preventing Argentine ant invasion in citrus orchards. Understanding these interactions helps us better protect our ecosystems and agricultural lands. By studying how native ants resist invasions, we can develop strategies to conserve biodiversity and maintain healthy ecosystems, benefiting both the environment and society in general.

**Abstract:**

The invasive Argentine ant (*Linepithema humile*) poses a significant threat to ecosystem stability worldwide. In Mediterranean citrus ecosystems, its spread may be limited by interactions with dominant native ant species. We conducted laboratory experiments to investigate the competitive dynamics between Argentine ants and two major native species, *Tapinoma nigerrimum* and *Lasius grandis*. At the individual level, both native species exhibited superior competitive performance, attributed to their larger body sizes and potential differences in chemical defences. At the colony level, *T. nigerrimum* demonstrated efficiency in interference competition, successfully defending food resources from Argentine ants. However, the Argentine ant exhibited higher recruitment capacity, albeit it was density-dependent. Our findings support the hypothesis that dominant native ants can serve as barriers against Argentine ant invasion in citrus ecosystems, highlighting the importance of interspecific competition in shaping community dynamics and invasive species management. This study underscores the potential role of native ant species in mitigating the impacts of invasive ants on ecosystem functioning and biodiversity conservation in agricultural landscapes, offering valuable insights for invasive species management strategies in Mediterranean citrus ecosystems.

## 1. Introduction

The Argentine ant, *Linepithema humile* (Mayr), stands out as a significant invasive species worldwide [1]. Originally from subtropical South America, it has been expanding its range to other regions since the nineteenth century [1]. Its invasion of the Mediterranean is believed to have begun in Portugal in the 1890s [2,3,4]. A recent review by Angulo et al. [5] provided valuable insights into the ecology of *L. humile*, synthesising its natural history, ecology, and impact as a successful invader. In particular, its traits promoting invasiveness are attributed to its diet, foraging and cooperative behaviour, effective recruitment, high reproductive rates, tolerance to diverse environments, and aggressiveness [5]. Thriving on an omnivorous diet that includes Hemipteran honeydew as a consistent source of carbohydrates, the Argentine ant alters ecosystems by outcompeting native ant species [6,7,8,9,10,11,12,13]. This invasive ant species demonstrates remarkable efficiency in rapidly recruiting large numbers of individuals, leading to the displacement of many native ant species [14]. Despite the Argentine ant’s capabilities, the success of its invasion may depend on competition with dominant native ant species [13,15].

It is important to understand how interspecific competition influences invaders’ success and simultaneously how a community of interacting species evolves in response to escalating rates of ecological change, such as the presence of invasive species, is a major challenge in ecology. Ant invasions offer valuable insights into ecological processes. For instance, studying the dynamics of ant invasions may reveal traits that promote behavioural or ecological dominance, aiding the prediction and prevention of the future establishment of invasive ant species [16]. While interspecific competition is recognized as a significant force shaping ant communities [9,17,18,19], its role remains debated [20,21]. Displacement of ant species from their habitat is the most severe outcome of interspecific competition [22], with various mechanisms such as interference and exploitation playing pivotal roles [18]. Interference competition occurs when a species asserts its dominance over a resource by either defending it from others or aggressively displacing ants already present at the resource [18,23]. Invasive ant species differ greatly in their performance in interference competition [24]. On the other hand, exploitative competition involves the ability of an ant species to locate a resource quickly by mobilizing a substantial number of workers to the resource before competitors can access it [18,23].

Within Mediterranean citrus ecosystems, the Argentine ant has emerged as a common pest [25]. Notably, a study conducted in southern Portugal revealed the absence of Argentine ants in the citrus orchards of one particular region, suggesting a potential role of native ant communities in resisting invasion [26]. Among other possibilities, we hypothesized that the composition of native ant community in that region may prevent the invasion by the Argentine ant. To explore this hypothesis, we employed two experimental approaches. Firstly, we compared the species diversity and frequency of ant assemblages foraging on the tree canopy in citrus orchards, distinguishing between invaded (treatment) and non-invaded (control) orchards by the Argentine ant. We assumed that any differences observed between treatment and control orchards in the structure and species composition of ant communities would result from interactions between native ants and the Argentine ant [27]. Additionally, in this study, we investigated interspecific competition through laboratory experiments, focusing on the behavioural dynamics between the Argentine ant and two major dominant ant species in Mediterranean citrus ecosystems, i.e., *Lasius grandis* Forel and *Tapinoma nigerrimum* (Nylander) [26].

In the present study, we aimed at testing the hypothesis that the composition of ant communities foraging on the citrus canopies in Mediterranean agroecosystems may prevent colonization by the Argentine ant, specifically through interactions with dominant native ant species such as *T. nigerrimum* and *L. grandis* [26]. To test this hypothesis, we conducted laboratory experiments using Petri dish arenas and cages. These experiments assessed the antagonistic interactions between the Argentine ant and the two dominant native ant species, at both individual and colony levels. Furthermore, we evaluated their performance in both interference and exploitative competition dynamics.

## 2. Materials and Methods

### 2.1. Origin and Maintenance of Ant Colonies

On 3 November 2016, one colony of each of the studied ant species, i.e., *L. humile*, *L. grandis,* and *T. nigerrimum,* containing more than 1000 workers, with queens and immatures, was collected from the rhizosphere of citrus orchards (*Citrus sinensis* L.) in the south of Portugal, Algarve (37°5′14.83″ N, 7°53′13.34″ W; 37°3′19.6992″ N, 7°57′2.5488″ W; 37°2′55.5288″ N, 7°56′53.07″ W, respectively). The orchards were selected based on prior knowledge of the geographical distribution of these species in the region [26]. Each nest yielded at least one queen and the maximum possible number or larvae, pupae, and workers. The collected individuals and nest soil were transported to the laboratory in containers coated with Fluon™ to prevent escape.

The obtained ant colonies were maintained in the laboratory under the controlled conditions of 21 ± 1 °C, 50–60% relative humidity within ventilated transparent acrylic cages (30–50 cm × 30 cm × 30 cm), and also being coated with Fluon™. They were kept in their original substrate and provided with water, a sugary solution, and a protein diet consisting of pollen, larvae of *Tenebrio molitor*, adults of *Drosophila* spp., boiled chicken eggs, and canned tuna.

The citrus mealybug, the *Planococcus citri* (Risso) (Hemiptera, Pseudococcidae) colony used in the bioassays as a honeydew food resource for the ants, originated from a population collected in citrus orchards, in Silves (37°12′ N, 8°18′ W; Portugal), in June 2006, and has since then maintained in the laboratory. Field-collected individuals were regularly added to the rearing to refresh the colonies. The insects were reared in climatic chambers (24 ± 0.5 °C, 60 ± 5% relative humidity, in total darkness), within plastic containers, and fed with sprouted potatoes (*Solanum tuberosum* L.).

### 2.2. Experiments

Ant interactions were investigated based on two different approaches. The first approach focused on individual interactions, where one individual from each ant species was exposed in three possible combinations: (1) *L. humile* × *T. nigerrimum*; (2) *L. humile* × *L. grandis*; and (3) *T. nigerrimum* × *L. grandis*. These experiments were conducted under laboratory conditions (average ± standard deviation of 22 ± 2 °C, 57 ± 6% relative humidity, and natural light), between November 2016 and June 2017. The second approach examined competition at the colony level between *T. nigerrimum* and *L. humile,* aiming to assess the capacity of *T. nigerrimum* to defend or invade a food resource in competition with *L. humile*. These experiments were conducted under laboratory conditions (23 ± 1 °C, 60 ± 9% relative humidity, and natural light) between April and May 2017.

#### 2.2.1. Individual Interactions

The experiment was conducted in glass Petri dish arenas (9 cm of diameter) coated with Fluon™. The objective was to investigate the outcome of competition between *L. humile* and the two native ant species, *T. nigerrimum* and *L. grandis*, at the individual level. Each trial involved the monitoring of the behavioural interactions between the two ants through direct observation and videotape recording for five minutes. Behavioural interactions were categorized based on a notation system adapted from Suarez et al. and Blight et al. [28,29]. This consisted in the following scheme: 1 = avoid, contacts that resulted in one or both ants retreating in opposite directions; 2 = contact, by antennation; 3 = aggression (biting, chemical defence, during less than two seconds); 4 = prolonged aggression, including death. An aggression score (*I*) was calculated for each species interaction based on the maximal aggression level that each species registered in each trial, according to the following formula:

(1) $$I=\left(-1\times f_{1}\right)+(1\times f_{2})+(2\times f_{3})+(3\times f_{4})$$

in which $f_{i}$ corresponds to the frequency of a particular type of behavioural interaction (*i* = 1, 2, 3, or 4) in each bioassay, as described before, adapted from [30].

Additionally, activity levels during the observation period were also registered, using the following notations: very active (2); active (1); no activity (0). After each assay, the outcome of each interaction was determined by assessing the status of individuals (healthy living, injured, moribund or dead) at 1 h and 24 h post-interaction. Subsequently, each specimen was preserved in ethanol 96% within Eppendorf tubes for posterior measurements. The body length of each individual was measured in millimetres under a stereomicroscope from the anterior edge of the head to the posterior corner of the gaster.

Each trial (ant species combination) was replicated 50 times, using different individuals and new Petri dishes. However, due to handling accidents (e.g., escape of individuals) only 40 replicates were validated for the interactions *L. grandis–T. nigerrimum* (LgT) and 38 for both *L. grandis*–*L. humile* (LgLh) and *L. humile*–*T. nigerrimum* (LhT). To evaluate species performance in each interaction experiment, a score was calculated considering the maximal aggression level, mean activity, and survival index at various time intervals post-interaction, according to the following equation:

(2) ∑(a+b+c)

with *a* = maximal aggression level; *b* = mean activity; *c* = survival index at 5 min, 1 h and 24 h (healthy living, injured, moribund, and dead individuals were notated as 3, 2, 1 and 0, respectively).

The species with the higher score was classified as the “winner”, i.e., the species showing the best performance in each replicate.

#### 2.2.2. Interaction between Colonies

Competition between *T. nigerrimum* and *L. humile* at the colony level was investigated by allowing each ant species access to a food resource in separate cages. Each ant species was allowed to access a food resource by connecting the ant colony cage (50 cm × 30 cm × 30 cm) to another cage with the food resource (30 cm × 30 cm × 30 cm), using a 20 cm clear flexible tube (20 mm diameter) to connect cages. The food resource consisted of a colony of *P. citri* (628 ± 88 individuals) installed on sprouted potatoes, offering a source of honeydew. The time taken for ants to find the exit tube, enter the food resource cage, and initiate foraging on the honeydew source was registered. The number of ants present in the food resource and the flow of ants within the tube were also estimated at different time intervals (30 min, 1 h, 1 h 30, 2 h, and 24 h). The ant flow estimate consisted of counting the number of individuals passing a virtual line in the middle of the tube, during one minute. Control trials were considered for each studied ant species (CA—control trial of *L. humile* and CT—control trial of *T. nigerrimum*).

After the colonization period, the food resource cage was connected to a third cage with the second ant species colony. Estimates of the same variables referred before for the first ant species were also obtained for the second species. The competition outcome was assessed by counting the number of dead, moribund, injured, and healthy living individuals of each ant species inside the food resource cage 1 h after exposure. Two types of trials were conducted based on the sequence of colonization of the food resource by the two ant species: (1) *T. nigerrimum* (1st species colonizing and defending the food resource) × *L. humile* (2nd species entering the resource cage, attacking 1st species and trying to claim the resource); and (2) *L. humile* × *T. nigerrimum*. The following notation was used to designate each trial, respectively: (1) T1Lh2; and (2) Lh1T2. Each trial was replicated five times with a recovery period of at least 48 h between trials to minimize potential impacts, as the same colonies were used for all the experiments.

### 2.3. Statistical Analysis

Statistical analyses were performed using Excel^TM^, SPSS for Windows and R [31,32]. Differences among species in interaction outcomes were analysed using χ^2^ tests. The result of the interaction for each replicate was based on the aggression score described before, i.e., the species with the best performance. A test was carried out for each observation period: 5 min, 1 h, and 24 h. A Multivariate Analysis of Variance (MANOVA) was performed to compare the means of different parameters (activity, maximal aggression level, and survival indexes) among species, and modalities (pairs of competing species). A Tuckey post hoc test was performed whenever significance of the parameter was exhibited. *t*-tests were used to access the differences between polymorphic classes of *T. nigerrimum* workers (minor workers with body length < 3.5 mm and major workers with body length > 3.5 mm) in relation to the studied parameters. We used a Levene test to test the homogeneity of variance; whenever this assumption was not fulfilled, we used an unequal variance *t*-test. Regarding the experiments at colony level, an ANOVA was used to assess the differences in the number of individuals at the resource cages, the individual flow of species and the time it took to arrive at the tube, the cage, and the resources. T-tests were used to access differences in the mean number of damaged, moribund, and dead individuals in the different trials.

## 3. Results

### 3.1. Individual Interactions

#### 3.1.1. Behavioural Interactions

The frequency of each behaviour varied among species and types of interactions. Avoidance was the most frequent behaviour, ranging between 23.7% and 50%, except for *T. nigerrimum* in interactions with *L. humile*, where aggression and prolonged fights or death were more common (Table 1). Antennation was the least frequent behaviour, occurring at rates of 0% to 2.6% in most interactions. All three ant species exhibited some degree of non-interaction (0–15.8%). *Linepithema humile* displayed a higher frequency of avoidance behaviour compared to the interacting species, while the opposite was observed for aggressive behaviour (Table 1). *Tapinoma nigerrimum* exhibited a higher frequency of aggression when interacting with *L. grandis*. The occurrence of prolonged fights or death ranged from 26.3% to 32.5%.

The mean maximal aggression level was 3.07 for *L. humile* versus *T. nigerrimum*, 3 for the *L. humile* versus *L. grandis* interaction, and 2.98 for the *L. grandis* versus *T. nigerrimum* interaction. *Tapinoma nigerrimum* exhibited the highest mean aggression levels and aggression scores, while *L. humile* displayed the lowest (Table 1). No significant differences were found among the three ant species in terms of maximal aggression level (Table 2).

#### 3.1.2. Activity

**Figure 1:**
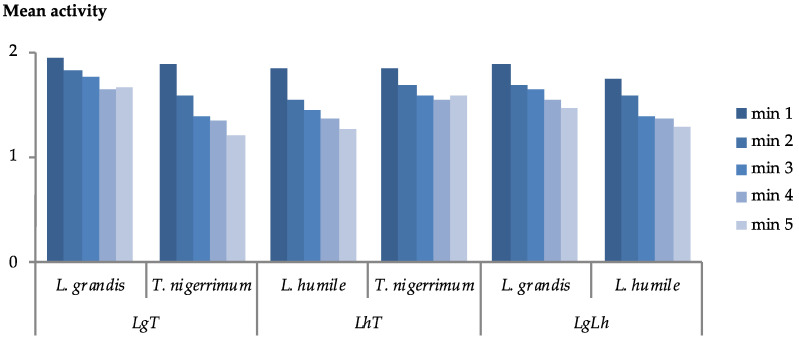
Variation of mean activity of *Lasius grandis* Forel, *Tapinoma nigerrimum* (Nylander), and *Linepithema humile* (Mayr) during each trial, and for each interaction (LgT, LhT, and LgLh).

The mean ant activity decreased over the 5 min trial period for all ant species and interactions (Figure 1). The decline in activity appeared to vary depending on the ant species and the specific interaction. For instance, *T. nigerrimum* exhibited a greater reduction in activity when paired with *L. grandis* compared to its interaction with *L. humile*. However, no significant differences were observed among ant species in terms of mean activity (Table 2).

#### 3.1.3. Survival

**Figure 2:**
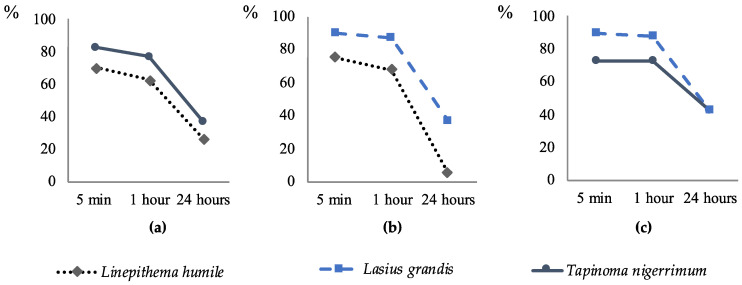
Survival rate (%) for each ant species at the end of trials at different time periods (5 min, 1 h, and 24 h after the beginning of the trials) according to the following interactions: (**a**) LhT—*L. humile* and *T. nigerrimum*; (**b**) LgLh—*L. grandis* and *L. humile*; (**c**) LgT—*L. grandis* and *T. nigerrimum*.

At the end of the 5 min bioassays, the survival rate of *L. grandis* was higher than that of *T. nigerrimum* and *L. humile* in both interactions (Figure 2). Conversely, *T. nigerrimum* exhibited a higher survival rate in the interaction with *L. humile* in the same period. The survival rates remained relatively stable up to 1 h after the bioassay for all three ant species but sharply declined after 24 h. Throughout this time period, the differences in survival rates between ant species remained consistent across all three species and interactions, except for LgT, where the survival rates of both *L. grandis* and *T. nigerrimum* converged to similar levels after 24 h (Figure 2). The survival index of *L. grandis* at 5 min, 1 h, and 24 h was significantly higher than that of *L. humile* (Table 2). No significant differences were observed between *L. grandis* and *T. nigerrimum* for all time periods, as well as between *T. nigerrimum* and *L. humile* at 5 min and 1 h. However, the survival index at 24 h for *T. nigerrimum* was significantly higher than that of *L. humile* (Table 2).

#### 3.1.4. Size Differences among Ant Species

Significant differences in body size were observed among ant species (F = 72.738, df = 2, *p* < 0.001, Table 2). The workers of *L. grandis* and *T. nigerrimum* were notably larger than those of *L. humile* (*p* < 0.0001, 95% C.I. = [0.69, 1.08] and *p* < 0.0001, 95% C.I. = [0.61, 1.00], respectively). However, no significant differences were found between the body sizes of *L. grandis* and *T. nigerrimum* (*p* = 0.57). No significant differences were observed among ant species in terms of mean activity (F = 2.87, df = 2, *p* = 0.06) and mean maximal aggression behaviour (F = 1.07, df = 2, *p* = 0.34). Survival index at 5 min, 1 h and 24 h was found to be significantly different among ant species (F = 6.69, df = 2, *p* < 0.01; F = 7.51, df = 2, *p* < 0.0001; and F = 7.60, *p* < 0.0001, respectively). *Lasius grandis* exibited significant higher survival index at 5 min 1 h and 24 h when paired with *L. humile* (*p* < 0.001, 95% C.I. = [0.15, 0.71]; *p* < 0.001, 95% C.I. = [0.22, 0.89]; and *p* < 0.001, 95% C.I. = [0.19, 1.21], respectively) but not when paired with *T. nigerrimum* (*p* = 0.19, *p* = 0.12, *p* = 0.95, respectively). *Tapinoma nigerrium* and *L. humile* exibited no differences concerning survival index at 5 min and 1 h (*p* = 0.14) but a different result occurred after 24 h (*p* = 0.001, 95% C.I. = [0.25, 1.27]), Table 2).

Additionally, body length varied significantly between major and minor workers of *T. nigerrimum* (t = 11.24, df = 76, *p* < 0.0001). No significant differences were found between these two size classes of ants concerning mean activity (t = 0.15, df = 76, *p* = 0.88), mean maximal aggression behaviour (t = 1.36, df = 76, *p* = 0.18), and survival index at 5 min (t = −0.65, df = 76, *p* = 0.52), 1 h (t = −0.40, df = 76, *p* = 0.70), and 24 h (t = −0.40, df = 76, *p* = 0.69) (Table 3).

#### 3.1.5. Outcome of the Interactions

**Figure 3:**
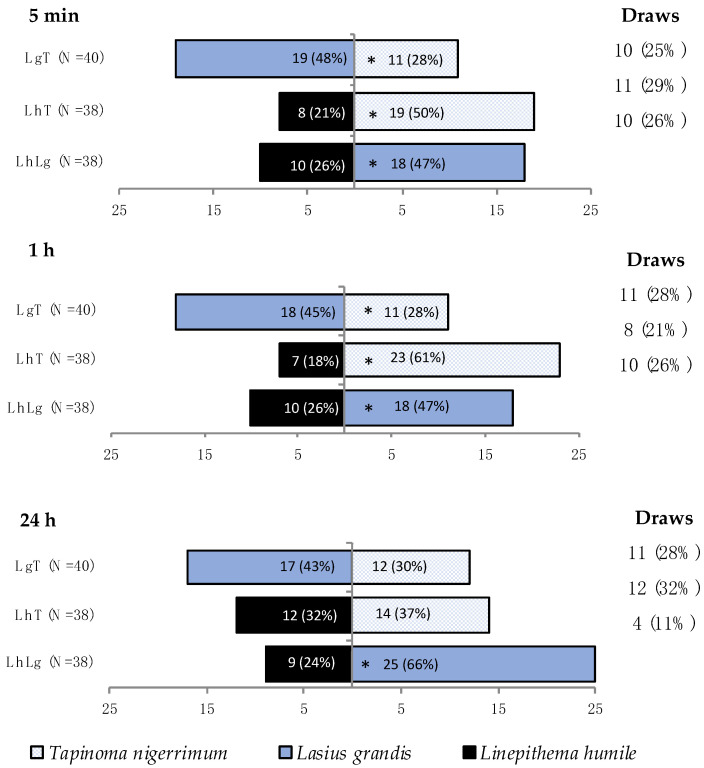
Outcome of the interaction among all three ant species, i.e., number of times a species emerged as the winner of the interaction according to scores calculated considering maximal aggression level, mean activity, and survival index at various time intervals post-interaction. LhLg = *Linepithema humile* (Mayr) vs. *Lasius grandis* Forel; LhT = *L. humile* vs. *Tapinoma nigerrimum* (Nylander); LgT = *L. grandis* vs. *T. nigerrimum*. Asterisks indicates significant differences between species for each interaction (*p* < 0.05). The number and percentage of draws is presented at the right side of the figure, for each interaction.

The outcome of the studied ant interactions was consistent for both 5 min and 1 h observations (Figure 3). *Lasius grandis* emerged as the winner in a significantly higher number of trials when interacting with both *L. humile* (χ^2^ = 6.041, df = 1, *p* = 0.014 for both 5 min and 1 h observations) and *T. nigerrimum* (χ^2^ = 5.263, df = 1, *p* = 0.022, for 5 min observations; χ^2^ = 3.959, df = 1, *p* = 0.047, for 1 h observations). The competition between *Tapinoma nigerrimum* and *L. humile* was won by the former species in a significantly higher number of trials (χ^2^ = 11.845, df = 1, *p* = 0.001 for 5 min observations; χ^2^ = 23.405, df = 1, *p* < 0.001 for 1 h observations). However, for the 24 h observations, only the interaction between *L. grandis* and *L. humile* showed significant differences (χ^2^ = 19.600, df = 1, *p* < 0.001). No significant differences were registered in the competition between *L. humile* and *T. nigerrimum* (χ^2^ = 0.362, df = 1, *p* = 0.547), as well as between *L. grandis* and *T. nigerrimum* (χ^2^ = 2.315, df = 1, *p* = 0.128) (Figure 3).

### 3.2. Interaction between Colonies

**Figure 4:**
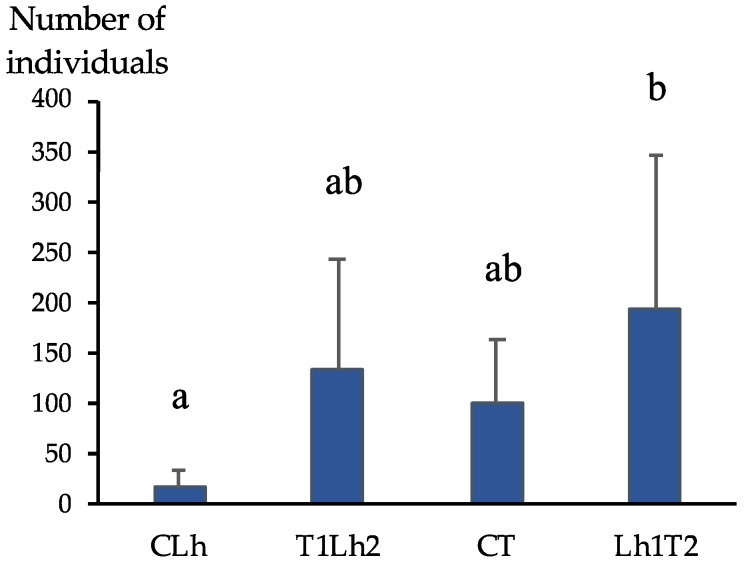
Mean number (and standard error) of individuals inside the resource cage after 1 h trial. CLh = Control trial of *Linepithema humile* (Mayr) (N = 8); T1Lh2 = *Tapinoma nigerrimum* (Nylander) defending the resource vs. *L. humile* claiming the resource (N = 5); CT = Control trial of *T. nigerrimum* (N = 8); Lh1T2 = *L. humile* defending the resource vs. *T. nigerrimum* claiming the resource (N = 5). Means followed by a different letter are significantly different (*p* < 0.05).

Both *L. humile* and *T. nigerrimum* registered a higher number of individuals in the resource cage when exposed to the other ant species, compared to the corresponding control trials (Figure 4). In attempts to invade the food resource defended by *T. nigerrimum, L. humile* recruited approximately 647% more individuals compared to the control. Conversely, in the opposite interaction, *T. nigerrimum* demonstrated around a 90% increase in the number of individuals recruited to the resource cage compared to the control. Additionally, the mean number of individuals in the control trials of *T. nigerrimum* was higher than that of *L. humile.*

**Figure 5:**
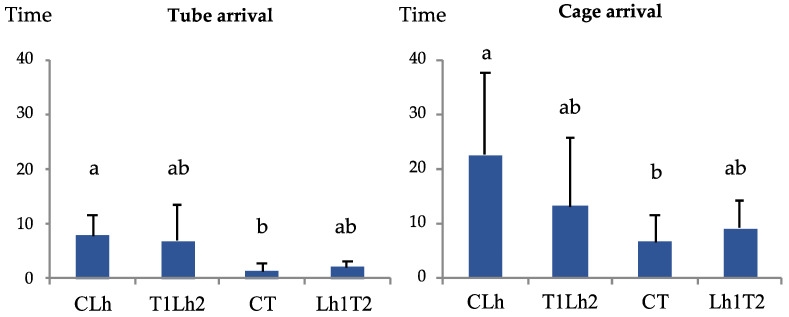
Mean time (minutes) (and standard error) of arrival to the tube and cage for each treatment and ant species control. CLh = Control trial of *Linepithema humile* (Mayr) (N = 8); T1Lh2 = *Tapinoma nigerrimum* (Nylander) defending the resource vs. *L. humile* claiming the resource (N = 5); CT = Control trial of *T. nigerrimum* (N = 8); Lh1T2 = *L. humile* defending the resource vs. *T. nigerrimum* claiming the resource (N = 5). Means followed by a different letter are significantly different (*p* < 0.05).

**Figure 6:**
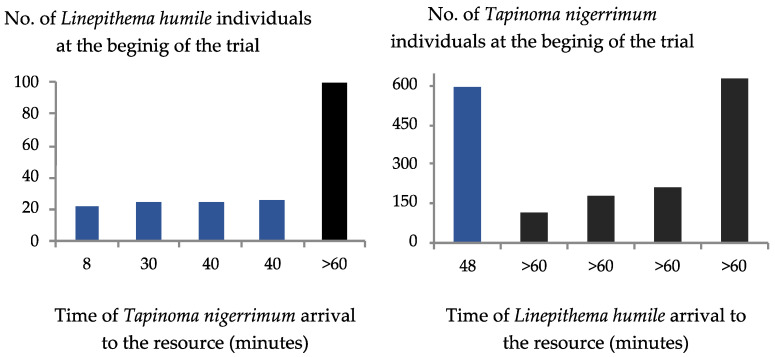
Time of arrival to the food resource (minutes) by *Linepithema humile* (Mayr) or *Tapinoma nigerrimum* (Nylander) in function of the initial number of individuals of the antagonist ant species defending the resource. Each column corresponds to a trial; blue columns means the species reach the resource while the black columns mean the species fail to reach the resource in the trial’s period of 1 h.

*Linepithema humile* took significant longer to enter the tube (*p* = 0.02, 95% C.I. = [−12.10, −0.83]) and cage (*p* < 0.05, 95% C.I. = [−31.88, −0.03]) than *T. nigerrimum* in the control trials (Figure 5). But no significant differences were found in the control trials compared to interactions with the other species or between interaction trials (Figure 5). In four out of five trials, *L. humile* failed to reach the food resource defended by *T. nigerrimum*, with only one individual managing to access the resource in that trial (Figure 6). In contrast, *T. nigerrimum* successfully colonized the food resource in all five trials, except for one instance where the initial number of Argentine ants was the highest (100 individuals, compared to a mean of 24 in the other replicates) (Figure 6). Often, *T. nigerrimum* actively fed on mealybug honeydew by the end of the trials and even 20–40 min before. At the beginning of the trials, the mean number of *T. nigerrimum* individuals was higher (mean number = 346, ranging from 114 to 630) than that of *L. humile* (mean number = 39.4, ranging from 22 to 100).

**Figure 7:**
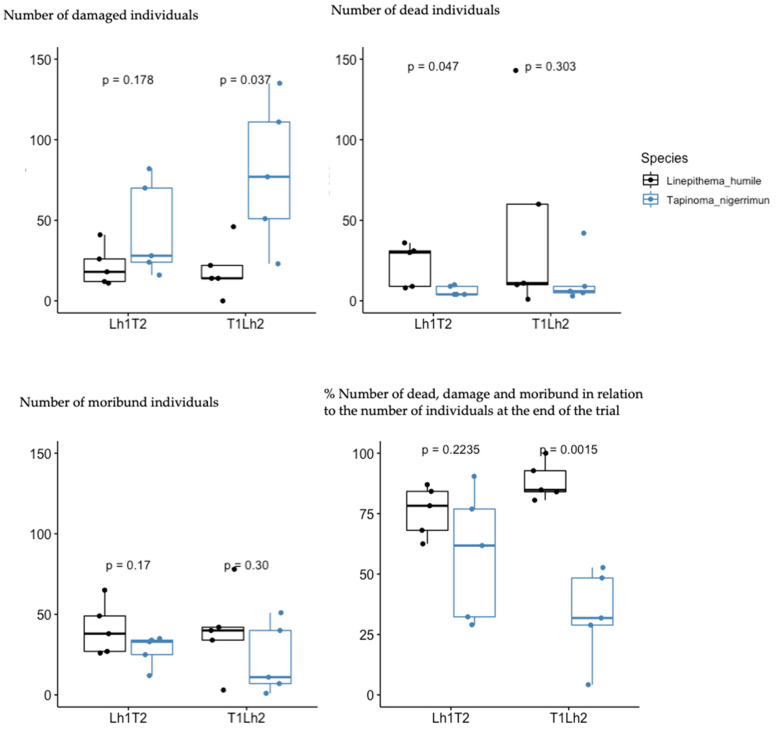
Number of damaged, moribund, and dead individuals for *Tapinoma nigerrimum* (Nylander) and *Linepithema humile* (Mayr) at the end of the trials, and percentage number of non-healthy living individuals in relation to the overall number of individuals at the end of the trial. T1Lh2 = *Tapinoma nigerrimum* (Nylander) defending the resource vs. *L. humile* claiming the resource (N = 5); Lh1T2 = *L. humile* defending the resource vs. *T. nigerrimum* claiming the resource (N = 5).

There were significant differences in the mean number of damaged individuals between species at the end of the T1Lh2 trials (t = −2.80, df = 5.12, *p* = 0.04), but not at the end of Lh1T2 (t = −1.55, df = 5.34, *p* =0.18), although values were notably higher for *T. nigerrimum* than *L. humile* in both treatments (Figure 7). On the other hand, there were significant differences in the mean number of dead individuals between species for Lh1T2 trials (t = 2.73, df = 4.42, *p* =0.047), but not for T1Lh2 (t = 1.16, df = 4.60, *p* = 0.30). The percentage (mean ± SE) of damaged, dead, or moribund individuals of *L. humile* (79.09 ± 4.72) in relation to the number of individuals at the end of the trials was significantly higher than that of *T. nigerrimum* (44.91 ± 7.38) for T1Lh2 trials (t = 5.97, df = 5.27, *p* =0.0015), but not for Lh1T2 (t = 1.39, df = 5.13, *p* = 0.22) (Figure 7).

**Figure 8:**
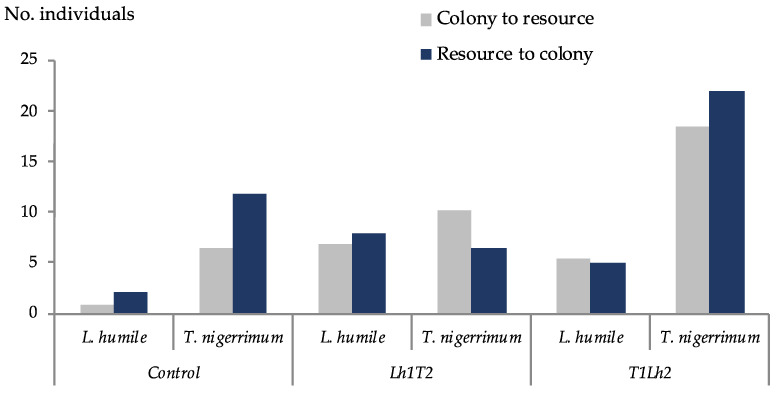
Mean number of individuals’ flow per minute within the tube at the end (1 h) of the trials for each species and treatment. CLh = control trial of *Linepithema humile* (Mayr) (N = 8); T1Lh2 = *Tapinoma nigerrimum* (Nylander) defending the resource vs. *L. humile* claiming the resource (N = 5); CT = control trial of *T. nigerrimum* (N = 8); Lh1T2 = *L. humile* defending the resource vs. *T. nigerrimum* claiming the resource (N = 5).

*Tapinoma nigerrimum* exhibited a significantly higher number of individuals’ flow per minute within the tube at the end (1 h) of the trials from colony to resource (F = 8.14, df = 1, *p* = 0.008) and from resource to colony (F = 8.36, df = 1, *p* = 0.007) than *L. humile* (Figure 8). Significant differences were found among treatments from colony to resource (F = 3.96, df = 2, *p* = 0.03), but only significant for T1Lh2 compared to the control (*p* = 0.03, 95% C.I. = [0.87, 15.68], (Figure 8)).

**Figure 9:**
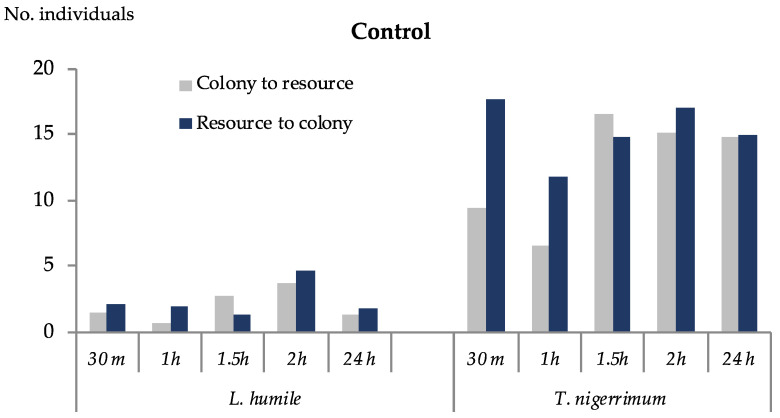
Mean number of individuals’ flow per minute within the tube between the colony and food resource cage for each ant species, in the control trials, during a period of 24 h.

The flow of individuals in the control between the colony and the food resource increased until 2 h after the beginning of the experiment for *L. humile* and 1.5 h for *T. nigerrimum* (Figure 9). This parameter remained at similar levels throughout the rest of the 24 h period for *T. nigerrimum* but decreased in *L. humile.* The mean values were consistently more than twice higher in *T. nigerrimum* than in *L. humile.*

## 4. Discussion

Field observations conducted by different authors have indicated that dominant native ants, including *L. grandis* [4], *T. nigerrimum* [13,15], *Pheidole pallidula* (Nylander), *Crematogaster scutellaris* (Olivier) [15], and *Iridomyrmex* spp. [33], may play a role in limiting, delaying or preventing the spread of Argentine ant invasions across different ecosystems. We hypothesized that this phenomenon might explain the absence of Argentine ants in citrus ecosystems within one of the three surveyed subregions of the Algarve (Serra), in southern Portugal [26]. In this study, we provide experimental evidence, derived from laboratory tests at both individual and colony levels, supporting our hypothesis that dominant native ant species, such as *T. nigerrimum* and *L. grandis,* can serve as formidable competitors against the invasive Argentine ant.

At the individual level, both *T. nigerrimum* and *L. grandis* demonstrated superior competitive performances compared to the Argentine ant, exhibiting higher levels of aggression and survival. This finding agrees with Frizzi et al. [34], which also observed a superior performance of *T. nigerrimum* against Argentine ants, in individual contests. The observed differences in body size between the two native ants and the Argentine ant may also contribute to this outcome, as both native species boast larger individuals. Previous research has highlighted the influence of body size on competitive ant interactions, exemplified by species such as *Cataglyphis niger* (Andre) [35] and *T. nigerrimum* [13]. The size differences between minor and major workers of *T. nigerrimum* did not seem to influence their activity, aggression level, or survival. Alternative metrics, such as mesosomal length [36] or head size [37] can be used as a proxy for body size and may offer more accurate categorization, since measurement is not affected by an enlarged gaster if the individual was recently fed. However, we used a classification method based on total body length that could allow a rapid screening of morphological variations and is usually highly correlated with head width [38]. Despite *T. nigerrimum* displaying greater aggression than *L. grandis,* the latter species revealed superior competitive performance overall. The competitive advantage observed in this Formicinae species may be partially attributed to potential differences in the impact of its chemical defences compared to those of the two Dolichoderinae species (*L. humile* and *T. nigerrimum*). In fact, Formicinae are known to spray venom secreted by their venom glands, whereas Dolichoderinae ants employ various chemical compounds, such as ketones and iridoids secreted by the pygidial glands [39]. Other studies have similarly shown that invasive ants often struggle in one-on-one competition with native ants [13] and references therein.

At the colony level, our experimental design allowed us to investigate interference competition between the Argentine ant and *T. nigerrimum. T. nigerrimum* proved to be more efficient than the Argentine ant, successfully defending a food resource from the Argentine ant in four out of five trials. However, in natural settings, the outcome of competitor interactions also depends on the exploitative competition. Our results provide only a partial insight into the exploitative competition between the two ant species. On one hand, *T. nigerrimum* exhibited faster food location compared to the Argentine ant. On the other hand, both ant species demonstrated the ability to recruit a relatively high number of individuals from their colonies when attempting to colonize a food resource defended by the competitor species. In relative terms, this recruitment capacity was about seven times higher in the Argentine ant than in *T. nigerrimum*. Nevertheless, the Argentine ant failed to access the food resource when it was defended by *T. nigerrimum*, whereas the opposite was true in most of the cases. The only occasion *T. nigerrimum* could not reach the food resource defended by the Argentine ant occurred when the latter species exhibited the highest number of individuals within the food resource cage during the trials. This suggests that the outcome of the interaction between *T. nigerrimum* and the Argentine ant may be density-dependent, a hypothesis supported by Holway et al. [40], who observed that the Argentine ant could maintain an average of 10 or more workers at bait sites in the presence of competitor ants, such as *Forelius mccooki* (McCook), only when its colonies exceeded 1000 workers. Nevertheless, in field conditions, the Argentine ant may exhibit a higher recruitment capacity due to its polygenic nature, with colonies comprising up to 16.3 queens per 1000 workers, each capable of producing up to 60 eggs per day. Additionally, the Argentine ant’s polydomous nest structure allows workers to move between nests, facilitating the recruitment of large numbers of individuals to locate and defend food resources [7].

Possible differences among ant species in attraction or preferences for food resources may also influence the outcome of competitive interactions. Grover at al. [41] found that sucrose deprivation reduces Argentine ant worker aggression and overall activity, but Frizzi et al. [34] showed that starvation had a scarce effect on individual aggressiveness in both *L. humile* and *T. nigerrimum*. Given that *T. nigerrimum* commonly feeds on aphid honeydew, while the Argentine ant is more frequently associated with other honeydew sources, including the citrus mealybug and other coccids [26], future studies should investigate the influence of different food sources on competitive interactions between these ant species.

Temperature conditions can also influence competitive interactions among ant species. For example, Frizzi et al. [34] provided experimental evidence, based on a laboratory study conducted at different temperatures (15, 20, 25, and 30 °C), that temperature may have a significant effect on competition performance in the interaction between the invasive garden ant, *Lasius neglectus* and two Mediterranean species, *C. scutellaris* and *T. nigerrimum.* The authors showed that the survival rate of each ant species during competition encounters was temperature dependent. Therefore, our results should be considered under the context of the experimental temperature conditions.

Finally, we must remember that, although ideally different colonies should be selected per ant species, as a source of individuals for the experiments on ant interaction, we decided to collect only one colony per ant species because (1) this experimental approach has been followed by other research, e.g., [42,43], and (2) collecting and maintaining several colonies for each of the three studied ant species in the laboratory was not practically feasible in our facilities, and also not recommended for conservation reasons (i.e., minimizing the impact on natural populations), at least in the case of the two native ant species.

## 5. Conclusions

In conclusion, our findings support the hypothesis that dominant native ant species, such as *T. nigerrimum* and *L. grandis*, may serve as barriers preventing the invasion of Mediterranean citrus ecosystems by the non-native Argentine ant. At the individual level, both native ant species exhibited higher levels of aggression and survival compared to the Argentine ant in interspecific interactions. However, it is crucial to distinguish between the individual and collective behaviours of ant populations [23]. In our experimental conditions, at the colony level, *T. nigerrimum* proved to be more efficient than the Argentine ant in competition, successfully defending food resources in four out of five trials against the former species’ attacks and dominating resources defended by the Argentine ant in four out of five trials.

## Figures and Tables

**Table 1:** Mean aggression level, aggression score, and frequency of each type of behaviour for each ant species and interaction in Petri dish bioassays.

Interaction (No. Replicates)	Ant Species	Frequency of Each Type of Behaviour (%)					Mean Aggression Level ± SE	Aggression Score
		0 = No Interaction	1 = Avoidance	2 = Antennation	3 = Aggression	4 = Prolonged Fight or Death		
LgT (N = 40)	*T. nigerrimum*	0.0	40.0	2.5	25.0	32.5	2.88 ± 0.14	110
	*L. grandis*	5.0	45.0	2.5	15.0	32.5	2.68 ± 0.18	85
LhT (N = 38)	*T. nigerrimum*	7.9	23.7	2.6	39.5	26.3	2.74 ± 0.18	137
	*L. humile*	15.8	31.6	0.0	26.3	26.3	2.47 ± 0.22	100
LgLh (N = 38)	*L. grandis*	10.5	34.2	0.0	29.0	26.3	2.61 ± 0.19	103
	*L. humile*	5.3	50.0	2.6	15.8	26.3	2.55 ± 0.18	63

**Table 2:** Mean size (length), mean activity, mean maximal aggression level, and survival index of the studied ants (mean ± SE).

Parameter ^1^		Ant Species	
	*Linepithema humile* N = 75	*Lasius grandis* N = 78	*Tapinoma nigerrimum* N = 78
Body size (mm)	2.81 ± 0.06 a	3.70 ± 0.06 b	3.62 ± 0.06 b
Activity	1.54 ± 0.06 a	1.71 ± 0.06 a	1.59 ± 0.06 a
Maximal aggression level	2.55 ± 0.13 a	2.64 ± 0.13 a	2.81 ± 0.13 a
Survival index 5 min	2.44 ± 0.08 a	2.87 ± 0.08 b	2.67 ± 0.08 ab
Survival index 1 h	2.27 ± 0.10 a	2.82 ± 0.10 b	2.54 ± 0.10 ab
Survival index 24 h	0.55 ± 0.15 a	1.24 ± 0.15 b	1.31 ± 0.15 b

^1^ Means followed by a different letter within a row are significantly different (*p* < 0.05).

**Table 3:** Body size (length), activity, maximal aggression level, and survival index of minor and major workers of *Tapinoma nigerrimum* (Nylander).

Variable	Size Class of Ant Workers	N	Mean ± SE
Body size (mm) *	Minor workers	37	3.03 ± 0.05
	Major workers	41	4.15 ± 0.08
Activity	Minor workers	37	8.35 ± 1.12
	Major workers	41	7.39 ± 1.01
Maximal aggression level	Minor workers	37	2.62 ± 0.18
	Major workers	41	2.98 ± 0.15
Survival index 5 min	Minor workers	37	2.73 ± 0.09
	Major workers	41	2.61 ± 0.13
Survival index 1 h	Minor workers	37	2.59 ± 0.14
	Major workers	41	2.49 ± 0.15
Survival index 24 h	Minor workers	37	1.05 ± 0.23
	Major workers	41	1.54 ± 0.23

* Variables marked with an asterisk showed significant differences between minor and major ant workers (*p* < 0.05).

## Data Availability

The raw data supporting the conclusions of this article will be made available by the authors on request.
